# Significance of neutrophil-to-lymphocyte ratio, platelet-to-lymphocyte ratio, lymphocyte-to-monocyte ratio and prognostic nutritional index for predicting clinical outcomes in T1–2 rectal cancer

**DOI:** 10.1186/s12885-020-6698-6

**Published:** 2020-03-12

**Authors:** Li-jian Xia, Wen Li, Jian-cheng Zhai, Chuan-wang Yan, Jing-bo Chen, Hui Yang

**Affiliations:** 1 Department of Colorectal and Anal Surgery, the First Affiliated Hospital of Shandong First Medical University, Jinan, 250012 Shandong Province China; 2 Department of Colorectal and Anal Surgery, Shandong Provincial Qianfoshan Hospital, Shandong University, Jinan, Jinan, 250012 Shandong Province China; 3 Department of Colorectal and Anal Surgery, Shandong Provincial Qianfoshan Hospital, Weifang Medical College, Jinan, 250012 Shandong Province China

**Keywords:** Rectal cancer, Inflammation, Prognosis, Complication

## Abstract

**Background:**

Inflammation-related parameters have been revealed to have prognostic value in multiple caners. However, the significance of some inflammation-related parameters, including the peripheral blood neutrophil-to-lymphocyte ratio (NLR), platelet-to-lymphocyte ratio (PLR), lymphocyte-to-monocyte ratio (LMR) and prognostic nutritional index (PNI), remains controversial in T1–2 rectal cancer (RC).

**Methods:**

Clinical data of 154 T1–2 RC patients were retrospectively reviewed. The cut-off values for NLR, PLR, LMR, and PNI were determined by receiver operating characteristic curves. The relationships of these parameters with postoperative morbidities and prognosis were statistically analysed.

**Results:**

The optimal cut-off values for preoperative NLR, PLR, LMR and PNI were 2.8, 140.0, 3.9, and 47.1, respectively. Significant but heterogeneous associations were found between NLR, PLR, LMR and PNI and clinicopathological factors. In addition, high NLR, high PLR, and low PNI were correlated with an increased postoperative morbidity rate. Patients with high NLR/PLR or low LMR/PNI had lower OS and DFS rates. On multivariate analysis, only high NLR was identified as an independent risk factor for poor DFS.

**Conclusions:**

NLR, PLR, and PNI are valuable factors for predicting postoperative complications in T1–2 RC patients. A preoperative NLR of more than 2.8 is an independent prognostic factor for poor DFS in T1–2 RC patients.

## Background

Colorectal cancer (CRC) is the fourth most common cancer and second leading cause of cancer-related death worldwide [1]. In 2018, more than seven hundred thousand people were diagnosed with rectal cancer (RC), and the overall mortality rate was 44.1% [1]. With the prevalence of health screening, more patients are diagnosed at a relatively early stage with less invasion depth. At present, the tumour-node-metastasis (TNM) staging system is the fundamental tool for predicting clinical outcomes and determining therapeutic options. The depth of invasion is associated with the prognosis of RC, particularly in the advanced stage. However, few reports have concentrated on investigating the predictive factors associated with prognosis for early T stage (T1–2) cancers [2]. Therefore, to develop more individualized treatment strategies for T1–2 RC patients, novel prognostic biomarkers that can be conveniently obtained preoperatively are needed [3, 4].

The pivotal role of the systemic inflammatory response in cancer progression has been well recognized and substantiated [5–7]. Peripheral blood cells might reflect the inflammatory and immune response of patients to malignant tumours and are critical for determining the treatment response and clinical outcomes of cancer patients. Inflammation-related parameters that evaluate the systemic inflammatory response have yielded prognostic value independent of the TNM staging system [8, 9]. Among these parameters, the peripheral blood neutrophil-to-lymphocyte ratio (NLR), platelet-to-lymphocyte ratio (PLR), lymphocyte-to-monocyte ratio (LMR) and prognostic nutritional index (PNI) [10] have been widely investigated, and their prognostic role has been demonstrated in various types of cancers, including RC [11–16]. However, most of these studies reported the prognostic value of these inflammation-related factors in locally advanced RCs [8, 14, 17, 18]. To the best of our knowledge, the prognostic significance of these factors in T1–2 RCs has been rarely reported, and the impact of these factors on postoperative complications remains obscure.

Our study aimed to detect the role of NLR, PLR, LMR, and PNI in predicting the prognosis of T1–2 RC patients without distant metastasis. Moreover, the association of these parameters with postoperative morbidity was investigated. In addition, the risk factors for poor survival in T1–2 RC patients were also analysed.

## Methods

### Patient cohort

We retrospectively reviewed 154 T1–2 RC patients who underwent R0 surgical resection between April 2012 and August 2016 at the First Affiliated Hospital of Shandong First Medical University. Magnetic resonance imaging was used to evaluate the clinical stage of the tumour preoperatively. The final diagnosis of the patients was confirmed by routine pathology. The exclusion criteria were as follows: recurrent or metastatic RC confirmed preoperatively or at surgery, emergency cases, unavailable clinicopathological data, more than 1 primary cancer, receiving anticancer treatments preoperatively, resections with macro- or microscopically positive pathological margins and with active infection or the use systemic corticosteroids. The TNM classification of malignant tumours, 8th edition, edited by the Union for International Cancer Control (UICC) was used to determine the TNM stage. Patients with T1 RCs and no signs of lymph node metastasis on endorectal ultrasound or MRI underwent local excision through transanal endoscopic microsurgery (TEM), or laparoscopic or open surgery was performed. Informed consent was obtained from each patient, and the present study was approved by the Ethics Committee of the Fist Affiliated Hospital of Shandong First Medical University.

### Definitions

Peripheral blood was obtained 1 week prior to surgery. The NLR was determined by dividing the absolute neutrophil count by the absolute lymphocyte count; the PLR was determined by dividing the absolute platelet count by the absolute lymphocyte count; and the LMR was determined by dividing the absolute lymphocyte count by the absolute monocyte count. The PNI was calculated by the following formula: serum albumin (g/L) + 5 × total lymphocyte count × 10^9^/L. [19] Postoperative complications were defined as any in-hospital or 30-day postoperative complication and graded according to the Clavien-Dindo classification [20].

### Follow-up and study endpoints

Patients were followed-up periodically after surgery. Re-examination was performed at 3-month intervals for the first 2 years postoperatively, every 6 months for the next 3 years and every year thereafter. Physical examinations and blood tests, including serum carcinoembryonic antigen (CEA) levels, were performed at each follow-up. A chest X-ray and abdominopelvic computed tomography scan were performed every 6 months, and colonoscopy was performed annually or when there was a suspicion of recurrence. In addition, rigid rectoscopy and endorectal ultrasound were conducted at every visit except for the colonoscopy visit of the TEM patients.

The primary endpoints were cancer recurrence or death. The secondary endpoint was the occurrence of postoperative complications. Overall survival (OS) was calculated as the date of diagnosis to the date of death from any cause. Disease-free survival (DFS) was defined as the time interval from cancer diagnosis until tumour recurrence or death from any cause.

### Statistical analysis

The data are presented as the mean ± standard deviation. Categorical variables were analysed with Pearson’s Chi-square test or Fisher’s exact test as appropriate. The cut-off values for NLR, PLR, LMR, and PNI were determined using receiver operating characteristic (ROC) curve analysis. At each ratio, the sensitivity and specificity for survival were determined and plotted, thereby generating a ROC curve. Using the (0, 1) criterion, the point on the curve with the shortest distance to the coordinate (0, 1) was chosen as the cut-off value, and the patients were classified into high and low NLR/PLR/LMR/PNI groups with this cut-off value. Kaplan–Meier analysis and the log rank test were used to compare the survival curves of the 2 groups. Risk factors for poor survival were detected by univariate and multivariate analyses using the Cox proportional hazards model. Variables with a *P* value of < 0.05 in the univariate analysis were further evaluated in the multivariate analysis to assess the independent predictors for OS and DFS. Statistical analyses were performed using the IBM SPSS statistics version 22.0 software package for Windows (IBM Co., New York, NY). A statistically significant difference was defined as a *P* value of < 0.05.

## Results

### Baseline patient characteristics and inflammatory-related parameters

**Fig. 1:**
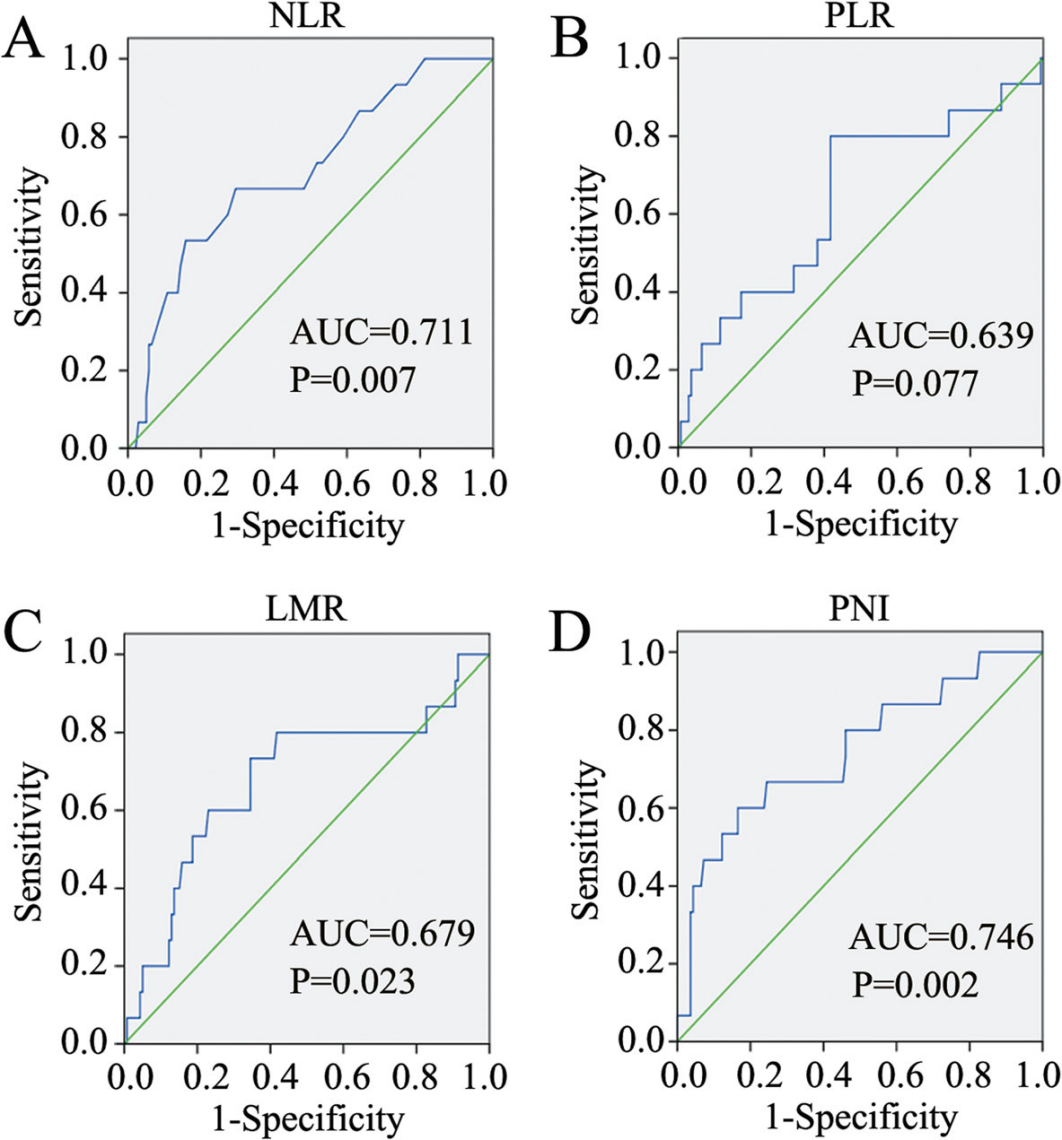
The cut-off values for the inflammation-related parameters. **a**-**d**. ROC curves were adopted to calculate the cut-off values for NLR (**a**), PLR (**b**), LMR (**c**), and PNI (**d**). NLR, neutrophil-to-lymphocyte ratio, PLR, platelet-to-lymphocyte ratio, LMR, lymphocyte-to-monocyte ratio, PNI, prognostic nutritional index, AUC, area of under curve

A total of 154 T1–2 RC patients were enrolled in this study, and lymph node metastasis was present in 22 patients. The characteristics of the patients are shown in Table 1. Our study group comprised 90 (58.4%) male and 64 (41.6%) female patients, with a mean age of 63.7 years (range 32–90 years). A total of 63 (40.9%) patients had 1 or more comorbidities. TEM was conducted in 47 patients, while laparoscopic (*n* = 53) or open surgery (*n* = 54) was performed in 107 patients. No mortality occurred 30 days after the operation. A total of 26 complications (grade I-IVa) occurred in 22 (14.3%) patients postoperatively, including 22 grade I-II and 4 grade III-IVa complications. With a median follow-up interval of 42.4 months (range 12–89 months), the 3-year OS and DFS rates of all patients were 90.9 and 87.7%, respectively. Three patients died from a cause other than rectal cancer. The distributions of preoperative inflammatory-related parameters are shown in Table 2. The optimal cut-off values for preoperative NLR, PLR, LMR and PNI that best predicted OS were calculated to be 2.8 (area under the curve (AUC): 0.71; sensitivity: 53.0%; specificity: 84.0%), 140.0 (AUC: 0.64; sensitivity: 80.0%; specificity: 58.0%), 3.9 (AUC: 0.68; sensitivity: 73.0%; specificity: 65.0%), and 47.1 (AUC: 0.75; sensitivity: 60.0%; specificity: 83.0%), respectively (Fig. 1a-d). Then, the patients were dichotomized into high or low NLR/PLR/LMR/PNI groups with these cut-off values. The numbers and features of patients in each group are listed in Table 1.

**Table 1:** Correlation between inflammatory parameters and clinicopathological characteristics

Parameters	NO. (154)	NLR	*P* value	PLR	*P* value	LMR	*P* value	PNI	*P* value
		Low (124) /High (30)		Low (84) / High (70)		Low (59) /High (95)		Low (32) /High (122)	
Age			0.054		0.622		0.558		0.441
≤ 60 years	54	48/6		28/26		19/35		8/46	
> 60 years	100	76/24		56/44		40/60		24/98	
Gender			0.308		0.720		0.063		0.184
Male	90	70/20		48/42		40/50		22/68	
Female	64	54/10		36/28		19/45		10/54	
Smoking			0.650		0.183		0.628		0.815
Yes	41	34/7		26/15		17/24		8/33	
No	113	90/23		58/55		42/71		24/89	
Alcoholism			0.780		0.079		0.720		0.962
Yes	39	32/7		26/13		14/25		8/31	
No	115	92/23		58/57		45/70		24/91	
Hypertension			0.917		0.382		0.373		0.555
Yes	45	36/9		27/18		16/29		8/37	
No	109	88/21		57/52		43/56		24/85	
Diabetes Mellitus			1.000		0.165		0.839		0.240
Yes	22	18/4		15/7		8/14		2/20	
No	132	106/26		69/63		51/81		30/102	
Coronary Artery Disease			0.153		0.247		0.056		0.216
Yes	21	14/7		9/12		12/9		7/14	
No	133	110/23		75/58		47/86		25/108	
CEA			0.415		0.593		0.023		0.261
< 5 μg/ml	126	103/23		70/56		43/83		24/102	
≥ 5 μg/ml	28	21/7		14/14		16/12		8/20	
CA19–9			1.000		0.127		0.515		1.000
< 37 U/ml	147	118/29		78/69		55/92		31/116	
≥ 37 U/ml	7	6/1		6/1		4/3		1/6	
HGB			0.063		0.012		0.854		0.013
≥ 110 g/L	143	118/25		82/61		54/89		26/117	
< 110 g/L	11	6/5		2/9		5/6		6/5	
Occult blood			0.967		0.169		0.190		0.831
Yes	128	103/25		73/55		52/76		27/101	
No	26	21/5		11/15		7/19		5/21	
Distance from anal verge			0.838		0.877		0.103		0.355
≤ 50 mm	54	43/11		29/25		16/38		9/45	
> 50 mm	100	81/19		55/45		43/57		23/77	
Operation procedure			0.945		0.010		0.149		0.446
TEM	47	38/9		33/14		14/33		8/39	
Radical resection	107	86/21		51/56		45/62		24/83	
Time of operation			0.645		0.067		0.125		0.165
< 3 h	56	44/12		36/20		17/39		15/41	
≥ 3 h	98	80/18		48/50		42/56		17/81	
Blood transfusion perioperation			0.024		0.626		0.973		0.191
Yes	4	1/3		3/1		1/3		2/2	
No	150	123/27		81/69		58/92		30/120	
Differentiation grade			0.195		0.118		0.458		0.349
Well+Moderate	126	99/27		65/61		50/76		28/98	
Poor+Undifferentiate	28	25/3		19/9		9/19		4/24	
Tumor size			0.003		0.575		0.111		0.764
< 3 cm	83	66/17		47/36		27/56		18/65	
≥ 3 cm	71	68/3		37/34		32/39		14/57	
T stage			0.389		0.952		0.052		0.255
T1	62	52/10		34/28		18/44		14/48	
T2	92	72/20		50/42		41/51		18/74	
N stage			0.901		0.064		< 0.001		0.096
N0	132	107/25		76/56		42/90		24/108	
N1/2	22	17/5		8/14		17/5		8/14	
TNM stage			0.901		0.064		< 0.001		0.096
I	132	107/25		76/56		42/90		24/108	
III	22	17/5		8/14		17/5		8/14	

*NLR* neutrophil-to-lymphocyte ratio; *PLR* platelet-to-lymphocyte ratio; *LMR* lymphocyte-to-monocyte ratio; *PNI* prognostic nutritional index; *CEA* carcinoembryonic antigen; *CA19–9* carbohydrate antigen 19–9; *HGB* hemoglobin; *TEM* transanal endoscopic microsurgery; *TNM* tumor-lymph node-metastasis

**Table 2:** Distribution of inflammation-related parameters in T1–2 rectal cancer patients

Parameters	Minimum value	Maximum value	Mean value	Standard deviation
Neutrophil count (10^9^/L)	0.06	9.44	3.63	1.39
Lymphocyte count (10^9^/L)	0.51	3.63	1.83	0.58
Platelet count (10^9^/L)	88.00	484.00	235.03	64.48
Monocyte count (10^9^/L)	0.12	0.97	0.43	0.16
Serum albumin (g/L)	32.10	53.40	42.90	4.32
NLR	0.02	12.26	2.22	1.32
PLR	32.60	403.00	140.77	56.86
LMR	1.06	16.69	4.76	2.27
PNI	37.75	65.95	52.06	5.40

*NLR* neutrophil-to-lymphocyte ratio; *PLR* platelet-to-lymphocyte ratio; *LMR* lymphocyte-to-monocyte ratio; *PNI* prognostic nutritional index

### Correlations between NLR, PLR, LMR and PNI and clinicopathological variables

To determine the clinical significance of NLR, PLR, LMR and PNI in T1–2 RC patients, the associations of NLR, PLR, LMR and PNI with clinicopathological features were analysed. The results showed that NLR was significantly correlated with perioperative blood transfusion (*P* = 0.024) and tumour size (*P* = 0.003) (Table 1). PLR was correlated with haemoglobin (HGB) level (*P* = 0.012) and TEM procedure (*P* = 0.010) (Table 1). In addition, LMR was significantly correlated with CEA level (*P* = 0.023), N stage (*P* < 0.001) and TNM stage (P < 0.001) (Table 1). PNI was correlated with only HGB level (*P* = 0.013) (Table 1). Distribution of inflammation-related parameters in T1–2 rectal cancer patients are listed in Table 2. Furthermore, the relationships of NLR, PLR, LMR, and PNI with postoperative complications were investigated. High NLR (*P* < 0.001), high PLR (*P* = 0.025), and low PNI (P < 0.001) indicated a much-increased morbidity rate postoperatively (Table 3). In addition, high NLR (*P* < 0.001) and low PNI (*P* = 0.005) were also correlated with higher rates of grade I-II complications (Table 3).

**Table 3:** Association between inflammation-related parameters and postoperative complications

Classifi-cation	No. of complications	NLR	P value	PLR	P value	LMR	P value	PNI	P value
		Low (124) /High (30)		Low (84)/ High (70)		Low (59) /High (95)		Low (32) /High (122)	
Grade I	2 postop bleed; conservative tx 3 urinary retention; catheterization 2 wound infection; opened at the bedside 2 non-infectious diarrhea; conservative tx	3/6	0.001	2/7	0.097	5/4	0.457	4/5	0.042
Grade II	4 postop bleed; blood transfusion 1 urinary tract infection; antibiotic tx 4 ileus, total parenteral nutrition 2 pneumonia; antibiotic tx 1 diarrhea; antibiotic tx 1 tachyarrhythmia; β-receptor antagonists tx	3/10	< 0.001	6/7	0.525	5/8	1.000	6/7	0.046
Grade I-II	22	6/16	< 0.001	8/14	0.064	10/12	0.457	10/12	0.005
Grade IIIa	2 stricture of the anastomosis; endoscopic dilatation	0/2	0.037	0/2	0.205	2/0	0.145	1/1	0.373
Grade IIIb	1 strangulating intestinal obstruction; reintervention	1/0	1.000	0/1	0.455	1/0	0.386	1/0	0.208
Grade IVa	1 anastomotic leak and postop bleed,; reintervention and intensive care unit	1/0	1.000	1/0	1.000	1/0	0.386	0/1	1.000
Grade III- IVa	4	2/2	0.171	1/3	0.488	4/0	0.040	2/2	0.191
Total	26	8/18	< 0.001	9/17	0.025	14/12	0.074	12/14	< 0.001

*NLR* neutrophil-to-lymphocyte ratio; *PLR* platelet-to-lymphocyte ratio; *LMR* lymphocyte-to-monocyte ratio; *PNI* prognostic nutritional index; *tx* treatment

### Survival analysis with NLR, PLR, LMR and PNI

**Fig. 2:**
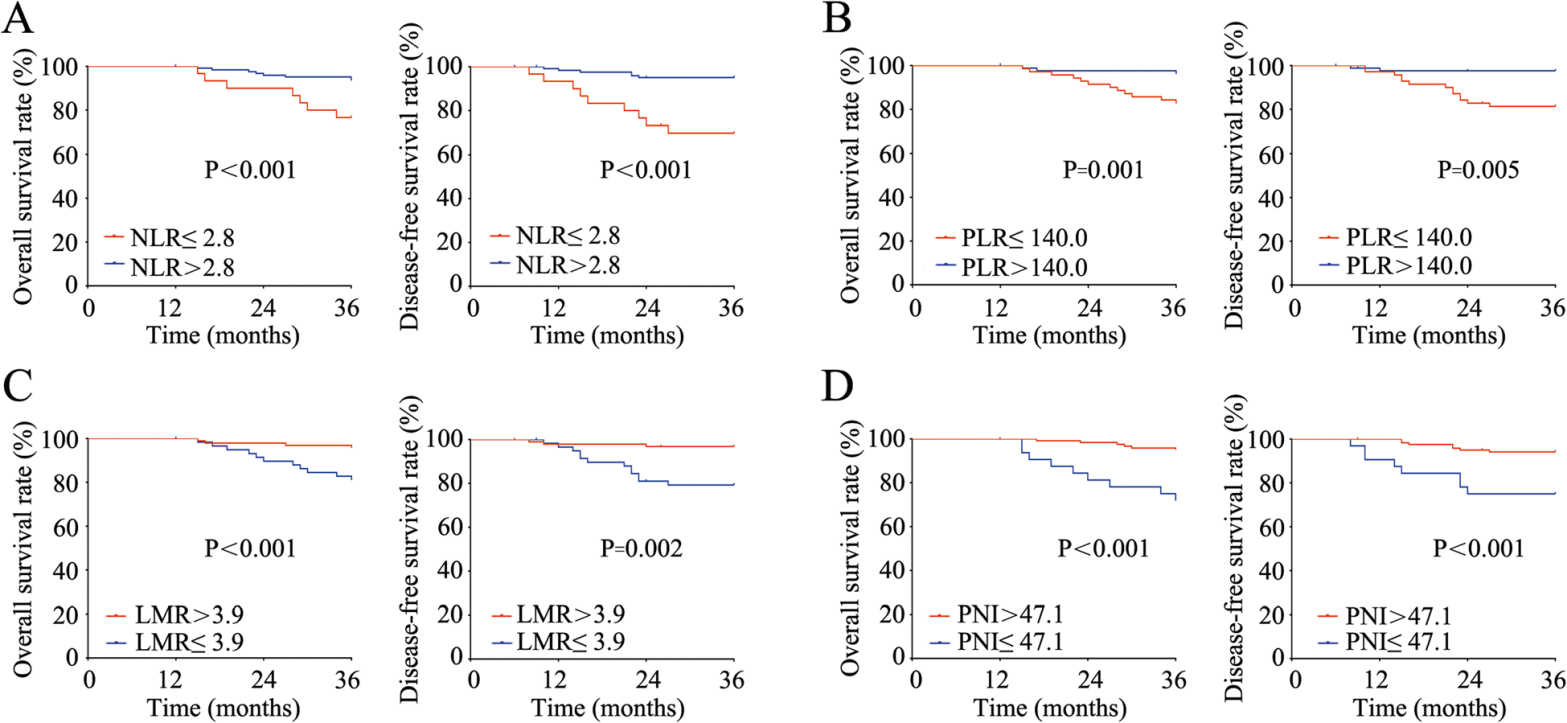
The associations of the inflammation-related parameters with the OS and DFS. **a**-**d**. The OS (left) and DFS (right) rates of T1–2 RC patients with high or low NLR (**a**), PLR (**b**), LMR (**c**), or PNI (**d**) level depicted by the Kaplan-Meier method. NLR, neutrophil-to-lymphocyte ratio, PLR, platelet-to-lymphocyte ratio, LMR, lymphocyte-to-monocyte ratio

To further define the value of the inflammatory-related parameters in predicting clinical outcomes in T1–2 RC patients, the OS and DFS rates of the patients in different subgroups were subsequently calculated. As displayed in Fig. 2, patients with high NLR, high PLR, low LMR, and low PNI showed a much worse 3-year OS rate than patients with low NLR (*P* < 0.001), low PLR (*P* = 0.001), high LMR (P < 0.001), and high PNI (P < 0.001). Moreover, patients with high NLR, high PLR, low LMR, and low PNI had much lower 3-year DFS rates than patients with low NLR (P < 0.001), low PLR (*P* = 0.005), high LMR (*P* = 0.002), and high PNI (*P* < 0.001) (Fig. 2a-d). Furthermore, the risk factors for poor OS and DFS were detected with univariate analysis, which showed that HGB < 110 g/L, high NLR, high PLR, low LMR, low PNI, more advanced N stage and TNM stage were risk factors for both poor OS and poor DFS (Table 4 and Table 5). To avoid multicollinearity, we conducted multivariate analysis using 2 models separately, and each multivariate model included either the N stage or TNM stage. Further subjecting these factors to multivariate analysis showed that only HGB < 110 g/L (*P* = 0.015), more advanced N stage (*P* < 0.001) and TNM stage (P < 0.001) were independent risk factors for poor OS (Table 4). HGB < 110 g/L (*P* = 0.014), high NLR (*P* = 0.009), more advanced N stage (P < 0.001) and TNM stage (P < 0.001) were independently associated with poor DFS (Table 5).

**Table 4:** Univariate and multivariate Cox regression analysis of the risk factor for poor overall survival

Parameters	Univariate analysis			Multivariate analysis		
	HR	95% CI	P value	HR	95% CI	P value
Age > 60 years vs. ≤60 years	1.510	0.481–4.744	0.480			
Gender Male vs. Female	2.918	0.823–10.341	0.097			
Smoking No vs. Yes	0.420	0.095–1.863	0.254			
Alcoholism No vs. Yes	0.734	0.207–2.600	0.631			
Hypertension Yes vs. No	2.198	0.797–6.063	0.128			
Diabetes Mellitus Yes vs. No	2.159	0.687–6.780	0.187			
Coronary Artery Disease Yes vs. No	2.550	0.812–8.010	0.109			
CEA level ≥ 5 μg/ml vs. < 5 μg/ml	2.380	0.813–6.966	0.113			
CA19–9 < 37 U/ml vs. ≥37 U/ml	0.046	< 0.001–1702.151	0.567			
HGB ≥110 g/L vs. < 110 g/L	0.172	0.055–0.542	0.003	0.204	0.057–0.731	0.015
NLR ≥2.80 vs. < 2.80	5.396	1.954–14.896	0.001	3.149	0.933–12.525	0.063
PLR ≥140.05 vs. < 140.05	5.043	1.423–17.874	0.012	1.266	0.277–5.783	0.761
LMR ≥3.88 vs. < 3.88	0.208	0.066–0.652	0.007	0.767	0.202–2.910	0.696
PNI ≥47.1 vs. < 47.1	0.152	0.054–0.427	0.295	0.462	0.127–1.686	0.242
Occult blood No vs. Yes	0.560	0.178–1.759	0.321			
Distance from anal verge ≤50 mm vs. > 50 mm	2.287	0.645–8.104	0.200			
Operation procedure Radical resection vs. TEM	1.823	0.514–6.461	0.352			
Time of operation ≥3 h vs. < 3 h	1.600	0.510–5.026	0.421			
Blood transfusion perioperation Yes vs. No	3.265	0.429–24.840	0.253			
Differentiation Poor/Undifferentiate vs. Well/Moderate	1.293	0.729–2.291	0.379			
Tumor size ≥3 cm vs. < 3 cm	1.409	0.511–3.886	0.508			
T stage T2 vs. T1	1.999	0.636–6.278	0.236			
N stage (N1/2 vs. N0)	11.888	4.215–33.532	< 0.001	9.944	3.001–32.954	< 0.001
TNM stage III vs. I	11.888	4.215–33.532	< 0.001	9.944	3.001–32.954	< 0.001

*CI* confidence interval; *HR* hazard ratio; *CEA* carcinoembryonic antigen; *CA19–9* carbohydrate antigen 19–9; *HGB* hemoglobin; *NLR* neutrophil-to-lymphocyte ratio; *PLR* platelet-to-lymphocyte ratio; *LMR* lymphocyte-to-monocyte ratio; *PNI* prognostic nutritional index; *TEM* transanal endoscopic microsurgery; *TNM* tumor-lymph node-metastasis

**Table 5:** Univariate and multivariate Cox regression analysis of the risk factor for poor disease-free survival

Parameters	Univariate analysis			Multivariate analysis		
	HR	95% CI	P value	HR	95% CI	P value
Age > 60 years vs. ≤60 years	1.480	0.471–4.649	0.502			
Gender Male vs. Female	2.025	0.645–6.362	0.227			
Smoking No vs. Yes	0.431	0.097–1.909	0.268			
Alcoholism No vs. Yes	0.463	0.105–2.025	0.311			
Hypertension Yes vs. No	2.128	0.772–5.870	0.144			
Diabetes Mellitus Yes vs. No	1.513	0.427–5.362	0.521			
Coronary Artery Disease Yes vs. No	2.567	0.817–8.066	0.107			
CEA level ≥ 5 μg/ml vs. < 5 μg/ml	2.464	0.842–7.211	0.100			
CA19–9 < 37 U/ml vs. ≥37 U/ml	0.046	< 0.001–1734.515	0.567			
HGB ≥110 g/L vs. < 110 g/L	0.178	0.057–0.560	0.003	0.205	0.058–0.721	0.014
NLR ≥2.80 vs. < 2.80	6.935	2.466–19.499	< 0.001	6.656	1.616–27.418	0.009
PLR ≥140.05 vs. < 140.05	8.074	1.822–35.790	0.006	1.689	0.313–9.109	0.542
LMR ≥3.88 vs. < 3.88	0.143	0.040–0.508	0.003	0.392	0.096–1.597	0.191
PNI ≥47.1 vs. < 47.1	0.206	0.075–0.568	0.002	1.169	0.308–4.435	0.818
Occult blood No vs. Yes	0.802	0.226–2.843	0.733			
Distance from anal verge ≤50 mm vs. > 50 mm	1.595	0.508–5.009	0.424			
Operation procedure Radical resection vs. TEM	1.812	0.511–6.423	0.357			
Time of operation ≥3 h vs. < 3 h	1.594	0.508–5.006	0.425			
Blood transfusion perioperation Yes vs. No	3.312	0.435–25.192	0.247			
Differentiation Poor/Undifferentiate vs. Well/Moderate	1.300	0.734–2.304	0.369			
Tumor size ≥3 cm vs. < 3 cm	1.869	0.665–5.252	0.235			
T stage T2 vs. T1	2.918	0.823–10.341	0.097			
N stage (N1/2 vs. N0)	11.143	3.955–31.400	< 0.001	9.193	2.665–31.712	< 0.001
TNM stage III vs. I	11.143	3.955–31.400	< 0.001	9.193	2.665–31.712	< 0.001

*CI* confidence interval; *HR* hazard ratio; *CEA* carcinoembryonic antigen; *CA19–9* carbohydrate antigen 19–9; *HGB* hemoglobin; *NLR* neutrophil-to-lymphocyte ratio; *PLR* platelet-to-lymphocyte ratio; *LMR* lymphocyte-to-monocyte ratio; *PNI* prognostic nutritional index; *TEM* transanal endoscopic microsurgery; *TNM* tumor-lymph node-metastasis

## Discussion

Systemic inflammation plays a pivotal role in cancer proliferation and metastasis by acting on the local tumour microenvironment [21, 22]. Accumulating evidence has indicated the prognostic value of inflammation-related factors in RC patients with different baseline characteristics and TNM stages [8, 9, 11, 23–28]. Our study evaluated the clinical significance of NLR, PLR, LMR, and PNI in T1–2 RC patients with or without lymph node metastasis. To define the prognostic value of these inflammation-related factors, a ROC curve was used to determine cut-off scores. As a result, the optimal cut-off values for NLR, PLR, LMR and PNI were identified as 2.8, 140.0, 3.9, and 47.1, respectively. Interestingly, the obtained cut-off values for NLR and PLR were relatively low compared with those reported in previous studies (NLR, range 3.0–5.0 [18, 29]; PLR, range 123.0–150.0 [18, 27, 30]), while the cut-off values for LMR and PNI were relatively high compared with those reported in previous studies (LMR, range 2.1–3.8 [12]; PNI, range 35.0–49.2 [16]). This finding may be due to the relatively early T stages of the RC patients in our study.

In the inflammatory response to cancer, neutrophils may directly interact with circulating tumour cells, serve as reservoirs for circulating vascular endothelial growth factor, and facilitate metastasis [31–33]. Lymphocytes usually function as pivotal tumour suppressors by inducing cytotoxic cell death and producing cytokines that inhibit cancer cell proliferation and metastatic activity [34, 35]. Elevated NLR, caused by lymphocytopenia and/or a high neutrophil count, may lead to a poor immune response to malignancy and an increased potential for tumour recurrence [33, 36–40]. Thus, NLR is recognized as an efficient inflammation-based prognostic parameter in solid tumours [29]. Platelets may release angiogenic and putative tumour growth factors in the inflammatory response, accelerate endothelial cell growth and promote cancer progression [11]. Elevated PLR has been demonstrated to have a significant association with poor prognosis in CRC [19]. Similar to lymphocytes, monocytes are also key immune cells in the inflammatory response [41]. In contrast to lymphocytes, monocytes promote the growth and survival of cancer cells by providing trophic factors and thus directly accelerate the progression of cancer [42–44]. Low preoperative LMR was a dominant poor prognostic factor in multiple types of cancers [14, 41, 45]. Nutrition status is a fundamental factor that can determine the outcome of treatment for cancer [46]. PNI, which is calculated according to serum albumin levels and peripheral lymphocyte counts, reflects both the nutritional status and immune status of the patient [10]. A low PNI score has been proven to be an indicator of poor prognosis in cancers [17, 47, 48]. The prognostic value of NLR has been reported in T1–2N0 CRCs [2], with the conclusion that preoperative NLR is a predictive prognostic factor for DFS and cancer-specific survival in patients with stage I CRC who underwent curative surgery. However, the predictive significance of PLR, NLR, and PNI for postoperative complications and prognosis has rarely been reported in T1–2 RCs. Impressively, our results revealed that T1–2 RC patients with high NLR/PLR or low LMR/PNI had much lower 3-year OS rates and DFS rates than patients with low NLR/PLR or high LMR/PNI. Moreover, high NLR/PLR and low LMR/PNI were all revealed as risk factors for poor OS and DFS in univariate analysis. However, these parameters were not identified as independent risk factors for poor OS in multivariate analysis, and only high NLR (HR = 6.656, 95% CI = 1.616–27.418, *P* = 0.009) was analysed as an independent risk factor for poor DFS, which is similar to the results reported by George Malietzis et al. in 2014 [49]. Overall, high NLR/PLR and low LMR/PNI can be used as indicators for poor OS and DFS in T1–2 RC patients with or without lymph node metastasis, and NLR may have extra significance independently of other factors in the prediction of DFS. Differentiating the patients with high risks of recurrence and poor survival in T1–2 RC patients may provide evidence for making a more rigid and personalized surveillance regimen.

Few studies have focused on the association of inflammation-related factors and postoperative complications in T1–2 RC patients. This study revealed that high NLR/PLR and low PNI were correlated with a higher morbidity rate. Moreover, high NLR and low PNI were also correlated with a higher grade I-II complication rate in subgroup analyses. In addition, there was a tendency towards an increased morbidity rate in patients with low LMR, though no statistical significance was found (*P* = 0.074). Thus, the inflammation-related factors may be used as markers for identifying patients with a high probability of occurring complications postoperatively, and more targeted treatment strategies should be made for these patients. Furthermore, significant but heterogeneous associations were found between the clinicopathological factors and the inflammation-related parameters. Previous studies have reported the association of lymph node metastasis with inflammation-related factors, but the results on the role of inflammation-related factors in predicting lymph node metastasis remain controversial [11, 49]. The present study discovered that LMR was the only factor correlated with N stage and TNM stage in T1–2 RC patients.

## Conclusion

The present study confirmed the value of NLR, PLR, LMR, and PNI in predicting postoperative complications and prognosis in T1–2 RC patients. However, only elevated NLR was identified as an independent risk factor for DFS. The ubiquity of complete blood count testing and the ease of calculation make these values ideal as predictive tools for clinical outcomes. However, this study has some limitations. The clinical data were retrospectively analysed, and the patients enrolled in this study were from one medical centre. In addition, the results of previous studies and our study have shown different cut-off values of the inflammation-related parameters in different TNM stages. Difference of cut-off value is a problem for clinical application. Prospective studies with more patients from multiple medical centres are needed in order to further verify the significance of NLR, PLR, LMR, and PNI in T1–2 RCs, and studies involving more samples with all TNM stages are also needed to create a model based on these inflammation-related parameters, which may facilitate the clinical application of these parameters.

## Data Availability

The datasets used and/or analysed during the current study are available from the corresponding author on reasonable request.

## Abbreviations

AUC: Area of under curve
CA19–9: carbohydrate antigen 19–9
CEA: Carcinoembryonic antigen
CRC: Colorectal cancer
DFS: Disease-free survival
HGB: Hemoglobin
LMR: Lymphocyte-to-monocyte ratio
NLR: Neutrophil-to-lymphocyte ratio
OS: Overall survival
PLR: Platelet-to-lymphocyte ratio
PNI: Prognostic nutritional index
RC: Rectal cancer
ROC: Receiver operating characteristic
TEM: Transanal endoscopic microsurgery
TNM: Tumor-lymph node-metastasis
TNM: Tumor-node-metastasis
